# The role of SGLT2i in attenuating residual cardiovascular risk through blood pressure-lowering: mechanistic insights and perspectives

**DOI:** 10.3389/fcdhc.2023.1243530

**Published:** 2023-09-26

**Authors:** Joaquim Barreto, Alessandra M. Campos-Staffico, Wilson Nadruz, Thiago Quinaglia, Andrei C. Sposito

**Affiliations:** ^1^ Laboratory of Atherosclerosis and Vascular Biology, University of Campinas (Unicamp), Campinas, Sao Paulo, Brazil; ^2^ Department of Clinical Pharmacy, College of Pharmacy, University of Michigan, Ann Arbor, MI, United States; ^3^ Cardiology Division, Clinics Hospital, Unicamp, Campinas, Sao Paulo, Brazil; ^4^ Massachussets General Hospital, Harvard University, Boston, MA, United States

**Keywords:** dapagliflozin, empagliflozin, cardiovascular risk, SGLT2i, residual risk

## Abstract

Sodium glucose cotransporter 2 inhibitors (SGLT2) have been increasingly pursued as a promising target for addressing residual cardiovascular risk. Prior trials demonstrated that SGLT2i not only promotes glucose-lowering, but also improves endothelial dysfunction, adiposity, fluid overload, and insulin sensitivity thus contributing to hemodynamic changes implicated in its cardiorenal benefits. The mechanisms in the effect of SGLT2i on blood pressure and their potential role in preventing cardiovascular events are hereby revised.

## Introduction

Cardiovascular disease remains the leading cause of death and disability in individuals with type 2 diabetes (T2D) (1). For a long time, achieving optimal glycemic control has been the primary goal of diabetes care due to robust evidence associating glucotoxicity with the progression of both micro- and macrovascular complications (2). However, recent findings have revealed that even with stricter glycemic control, the cardiovascular risk in T2D does not parallel that of healthy individuals (3, 4). The persistence of this risk, despite all the therapeutic interventions, is referred to as the residual cardiovascular risk. This is attributed to the complex, multi-faceted pathophysiology of T2D, which includes not only glucotoxicity but also other risk factors such as hypertension, adiposity, impaired cardiac energy metabolism, dyslipidemia, and kidney failure (5).

As a consequence, the development of new therapeutic targets that address these detrimental pathways alongside glucose-lowering has emerged as an encouraging approach to mitigate residual cardiovascular risk (6). This review provides a comprehensive exploration of the novel mechanistic insights that highlight the significant role of sodium-glucose cotransporter-2 inhibitors (SGLT2i) in attenuating residual cardiovascular risk in T2D. Specifically, we shed light on their blood pressure lowering effect, thereby positioning SGLT2i as reliable therapeutic agents in mitigating cardiovascular complications associated with T2D.

### Hypertension

**Figure 1:**
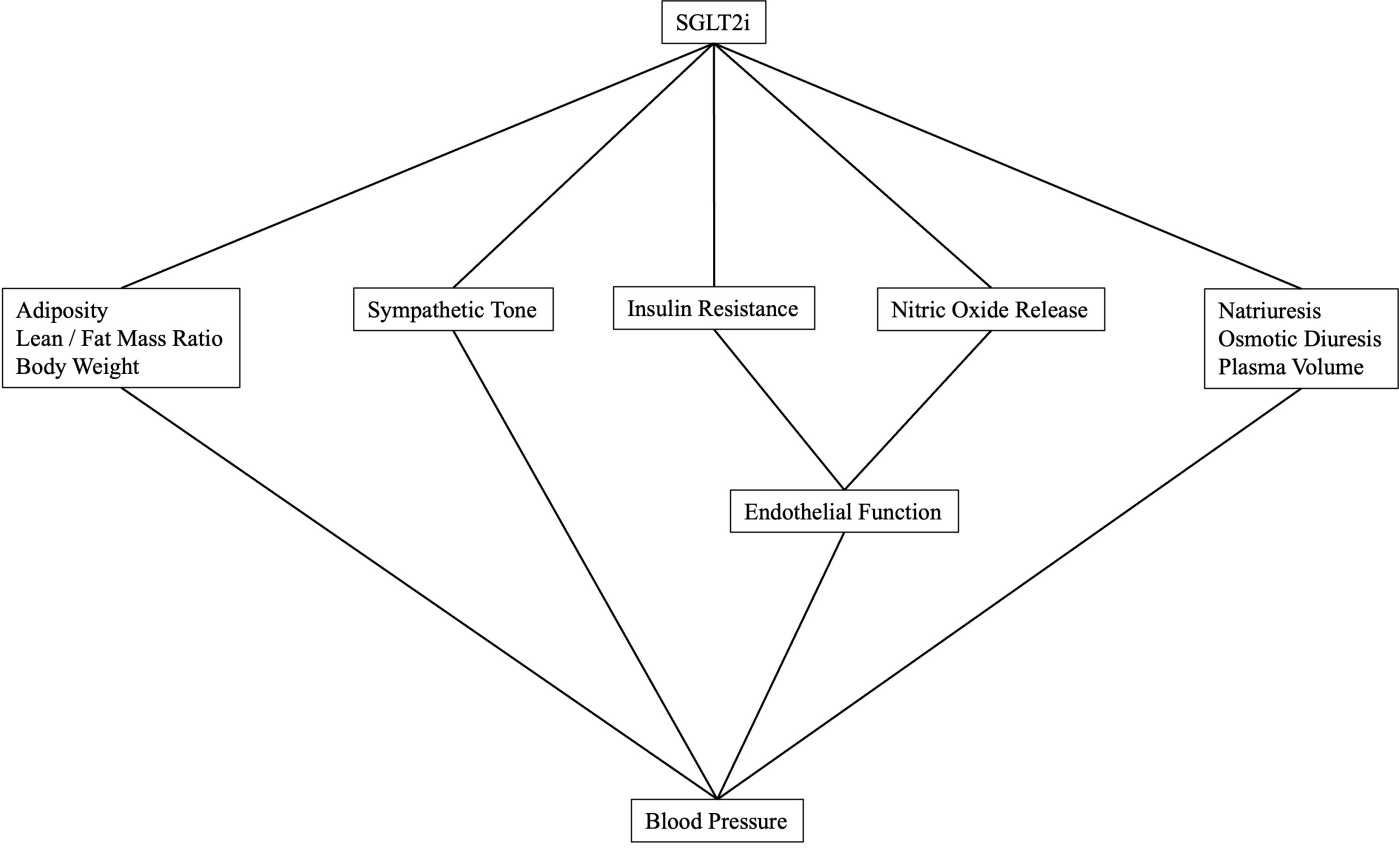
Mechanisms involved in blood pressure reduction by SGLT2i.

Hypertension is highly prevalent among individuals with T2D, significantly increasing the risk of cardiovascular disease on top of an already heavy burden (7, 8). Considering that both conditions overlap risk factors and pathophysiological pathways, it is estimated that half of individuals with hypertension have insulin resistance, and up to 85% of individuals with T2D also have hypertension (9). Experimental studies have shown that high blood pressure upregulates pathways related to insulin resistance, while T2D triggers the overactivation of the renin-angiotensin-aldosterone system and sympathetic tone, resulting in elevated blood pressure (7, 10). The interplay between T2D and hypertension synergically worsens cardiovascular risk (11, 12). Accordingly, individuals with both conditions have a twofold increase in cardiovascular mortality compared to those with only T2D or hypertension, and this effect linearly grows as blood pressure rise (13, 14). Promisingly, SGLT2i have demonstrated the ability to lower blood pressure whilst optimizing glycemic control (**Figure 1**).

In comparison to placebo, SGLT2i reduces 24-hour systolic and diastolic blood pressures by an average of 4 mmHg and 2 mmHg, respectively. This reduction remains consistent regardless of baseline blood pressure or changes in weight (15, 16). Given that a 10 mmHg drop in systolic blood pressure is estimated to decrease incident cardiovascular events by 12%, the impact of SGLT2i on blood pressure could significantly reduce morbidity (17, 18). Furthermore, despite evidence supporting stricter blood pressure targets for individuals at higher cardiovascular risk is beneficial for preventing adverse events, real-world data shows that less than a third of individuals with T2D meet these targets (19–21). SGLT2i could enhance the effects of concomitant antihypertensive drugs, thus improving compliance with blood pressure goals and reducing the incidence of adverse outcomes (20, 22).

### Osmotic diuresis

Until recently, blood pressure reduction through SGLT2i was attributed solely to osmotic diuresis. To investigate this further, Heerspink et al. (23) conducted a study on well-controlled individuals with T2D and examined the effect of 12 weeks of dapagliflozin treatment on radiolabeled-estimated plasma volume (23). The results showed that dapagliflozin led to a 7% decrease in plasma volume and a 10 mmHg reduction in 24-hour systolic blood pressure compared to the placebo group (23). While these early hemodynamic changes may be influenced by this mechanism, compensatory renal responses occurred due to plasma volume changes, ultimately restoring homeostasis (24).

In a related study, Scholtes et al. (24) explored the effects of dapagliflozin treatment on individuals with T2D on standardized sodium intake regimens. They found that dapagliflozin upregulated multiple water-conservation compensatory pathways, including vasopressin-mediated water retention, hyperactivation of the renin-angiotensin-aldosterone system, and a sharp decrease in fractional urea excretion (24–26). Consequently, renal adaptative responses play a significant role in mitigating the impact of volume contraction on SGLT2i-induced hemodynamic changes, suggesting the involvement of other concurrent pathways.

### Sympathetic tone

Adrenergic overdrive plays a pivotal role in the pathogenesis of hypertension and the progression of end-organ damage (27). In individuals with hypertension, the presence of T2D intensifies the hyperactivation of sympathetic tone, leading not only to elevated blood pressure but also an increased risk of left ventricle hypertrophy, myocardial infarction, heart failure, stroke and mortality (28–31). A study by Hamaoka et al. (32) demonstrated that a 12-week treatment with dapagliflozin in patients with heart failure resulted in a decrease in muscle sympathetic nerve activity and resting heart failure whilst reducing blood pressure. Similarly, Balcıoğlu et al. (33) found that a 24-week treatment with dapagliflozin improved heart rate variability and prevented ventricular premature beats, whereas SGLT2i therapy also prevented vaso-vagal syncope in the SCAN study (34).

These findings suggest that SGLT2i modulation of the autonomic nervous system may contribute to blood pressure reduction and potentially attenuate residual cardiovascular risk. However, further investigation is necessary to gain a comprehensive understanding of the underlying mechanisms of these drugs.

### Insulin resistance and endothelial dysfunction

Insulin resistance and endothelial dysfunction, which are key contributors to cardiovascular risk, are associated with increased blood pressure that could be prevented through the use of SGLT2i (35). Insulin plays a pivotal role in regulating blood pressure through two pathways: (i) the phosphatidylinositol 3-kinase (PI3K)/protein kinase-B (Akt)/nitric oxide (NO) pathway, which promotes vasodilatation and, ultimately, decrease blood pressure; and (ii) the mitogen-activated protein kinase (MAPK)/endothelin-1 (ET-1) pathway, which causes vasoconstriction and elevates blood pressure (36). In the presence of insulin resistance, the activity of the MAPK/ET-1 pathway dominates over that of the PI3K/Akt/NO pathway, leading to vasoconstriction with elevated blood pressure prevailing (36). Furthermore, insulin resistance has also been associated with hyperactivated sympathetic tone, activation of the renin-angiotensin aldosterone system, and increased salt-sensitivity; all of which are implicated in hypertension (12, 37–40).

Previous studies have demonstrated that SGLT2i therapy improves endothelial function and insulin sensitivity (41). For instance, in the ADDENDA trial, an open-label prospective randomized clinical trial that enrolled 98 individuals with T2D and carotid atherosclerotic disease, the efficacy of dapagliflozin treatment was examined over a 12-week period. The results demonstrated significant improvements in vasomotor function and increased plasma nitrite levels (42). This study adds to existing evidence that SGLT2i possess multiple beneficial effects. They have been shown to reduce oxidative stress, modulate adhesion molecules, and exhibit anti-inflammatory and anti-apoptotic properties. These collective effects ultimately contribute to the restoration of endothelial function (43–45).

SGLT2i also improve insulin sensitivity. A study by Merovci et al. (46) showed that 14 days of dapagliflozin therapy led to significant improvements in insulin-stimulated glucose disposal in individuals with T2D, suggesting increased muscle insulin sensitivity (46). Glycosuria was partially compensated by an increase in endogenous glucose synthesis following increased glucagon levels (46). Accordingly, Rodriguez et al. found that 12-week treatment with dapagliflozin improved insulin sensitivity, as assessed by serial measurements of plasma insulin and glucose levels adjusted for urinary glucose excretion, in a group of 24 adults with pre-diabetes undergoing oral glucose tolerant test (47). Although the mechanisms involved in SGLT2i abrogation of insulin resistance remains under debate, prior experiments suggest that SGLT2i prevention of glucotoxicity-induced oxidative stress of beta-cells may partially answer for this effect (48).

A recent meta-analysis with 55 clinical trials and over 36 thousand patients demonstrated that all SGLT2i consistently reduce serum uric acid (SUC) levels, compared to placebo, and this may contribute to insulin resistance and endothelial dysfunction reversion (49). In this regard, prior experiments showed that induction of hyperuricemia in T2D mice not only abrogate nitric oxide release and increased blood pressure, but also inhibited the expression vascular adhesion cell molecule *in vitro* (50). Furthermore, data from clinical studies support hyperuricemia as an independent risk factor not only for cardiovascular events, but also for rapid decline in kidney function of T2D individuals (51). Together, SGLT2i effect on SUC may hence contribute to the reduction of blood pressure and assist in overall cardiovascular risk reduction (52).

### Weight loss

SGLT2i therapy promotes weight loss, which also helps reducing residual cardiovascular risk. Currently, eight out of ten individuals with T2D are affected by excess weight (53). Addressing these conditions not only improves blood pressure and glycemic control, but also provides significant benefits in terms of peripheral neuropathy symptoms, exercise tolerance and overall quality of life (54–56). Studies have shown that a decrease of 1 unit in body mass index leads to a 5 mmHg reduction in systolic blood pressure, and a 10% reduction in body weight results in a 1% decrease in glycated hemoglobin (55, 57). Previous research has also demonstrated that a majority of hypertension cases are attributed to excess adiposity, and that reducing adiposity may lead in some cases to the remission of hypertension (58). While the complex relationship between obesity and hypertension is beyond the scope of this article (59), we will focus on the evidence implicating SGLT2i-induced weight loss on blood pressure changes.

In a recent meta-analysis of 18 randomized controlled trials enrolling over 1,430 participants, SGLT2i therapy was associated with an average weight reduction of 2.73 kg (60). Participants who were concomitantly on a regular exercise regimen and had preserved kidney function were twice as likely to experience a weight loss greater than 3% of their baseline weight, and these reductions were independently correlated to systolic blood pressure drops (61–63). Further studies conducted by our group demonstrated that weight loss primarily stems from fat mass loss. This finding is supported by the evidence of an increase in the lean-to-total mass ratio in individuals with T2D treated with dapagliflozin compared to those treated with glibenclamide alongside metformine in the ADDENDA trial (64). Other trials have also shown that the majority of total body mass change consists of water and fat mass loss, with minimal impact on lean mass loss (65–68).

The effect of SGLT2i on glycosuria is involved in adiposity reduction. In this matter, Rajeev et al. found that dapagliflozin increase daily urinary glucose excretion by up to 40g, which in turn translates in an incremental daily energy expenditure of 300kcal (69). The expected weight loss for such caloric deficit is of 10kg per year, which is higher than the observed change in body weight. Such discrepancy is partially explained by the activation of compensatory mechanisms that increase endogenous synthesis of glucose and appetite. In fact, earlier studies showed that dapagliflozin glucose-lowering effect is partially attenuated by a concomitant increase in glucagon and glucose synthesis of 32% and 17%, respectively, that may attenuate caloric deficit attenuating weight loss (46).

Seminal hypothesis-generating experimental studies have also been conducted. For example, Xu et al. showed that empagliflozin reduces inflammation in adipose tissue, increases energy expenditure and heat production, and promotes the expression of uncoupled protein 1 in brown fat tissue (70). Moreover, empagliflozin enhanced the beta oxidation of fatty acids through the upregulation of MAPK pathways, reduced liver steatosis, and increased adiponectin levels in ApoE^-/-^ mice (71). These effects may be abrogated by compensatory increased appetite. In fact, Devenny et al. (72) showed that dapagliflozin induces a 30% increased caloric intake in T2D mice with *ad libidum* access to food, thus resulting in a 3-fold lower body weight loss compared to animals with restricted access to diet (72). Beyond those mechanisms, the prevention of mitochondrial dysfunction and the modulation of adiponectin levels may be potential mechanisms underlying the impact of SGLT2i on adiposity, although those hypothesis warrants further verification (73, 74).

There has been a growing body of evidence from both clinical and experimental settings supporting the role of SGLT2i as a promising target for tackling overweight and obesity thus contributing to improved blood pressure control. So far, it has become clear that SGLT2i not only reduces body weight, but also shifts body composition toward a decrease in body fat percentage, promoting less atherogenic and hypertensive phenotypes, hence abrogating residual cardiovascular risk (65, 75).

## Genetic variants

The influence of gene polymorphisms on SGLT2i has been scarcely explored. For example, UDP-glucuronosyltransferase 1-9 (UGT1A9) enzyme answers for dapagliflozin glucuronidation to its inactive metabolite, dapagliflozin-3-O-glucuronide (D3OG). Prior studies demonstrated that UGT1A9 gene polymorphisms modulate UGT1A9 enzyme activity, and this may have repercussions on dapagliflozin pharmacodynamics, thus affecting its influence on several pathways implicated in blood pressure control (76–78). Furthermore, Solini et al. (79) demonstrated that dapagliflozin prompts putative epigenetic modulation of miR30e-5p and miR199a-3p, that in turn abrogate renal resistive index and may therefore contribute to a favorable hemodynamic profile potentially contributing to cardiovascular prevention (79).

Other *in vitro* experiments also support a role for canagliflozin in modulating sirtuin 3 gene expression in proximal tubular epithelial cells promoting both renoprotective as a potential driver of blood pressure lowering (80). Likewise, Zhao et al. showed that epigenetic modulation of transient receptor potential channel 3 prevented high-salt diet-induced hypertension in rats, and this effect was at least partially explained by parallel activation of TRPC3/NCX1 pathway modulating intracellular calcium handling (81). From this data, it is possible that individuals with salt‐sensitive hypertension, which is tightly regulated by gene polymorphisms, such as HSD11B2 and CYP3A5, involved in salt-sensitive hypertension, may experience greater blood pressure changes compared to non-salt-sensitive (82).

## Cardiorenal benefits

Prior trials provided solid evidence of cardiorenal benefits from SGLT2i treatment in high-risk T2D individuals. In the DECLARE TIMI 58 study dapagliflozin yielded a 17% (HR 0.87, 95%CI: 0.73 – 0.95; p= 0.005) relative risk reduction in the composite outcome of CV death or hospitalization for heart failure in 17,160 patients followed for 4.2 years, when compared to placebo (83). Likewise, in the CANVAS trial, canagliflozin reduced the relative risk of MACE by 14%, (HR 0.86; 95% CI: 0.75 – 0.97, p < 0.001), and that of renal outcomes including 40% reduction in glomerular filtration rate, renal replacement therapy and renal death, by over 40% (HR 0.60, 95% CI: 0.47-0.77). In line with these results, empagliflozin reduced the incidence of the composite outcome of CV death, myocardial infarction, and stroke by 14% (HR 0.86, 95% CI 0.74-0.99; p= 0.04), and that of hospitalization for heart failure by 35% (HR 0.65, 95% CI 0.50 – 0.85; p= 0.002) (84).

Renal benefits from SGLT2i in T2D subjects have been also explored in further trials. In this regard, the CREDENCE study was stopped early at 2.6 years after demonstrating a 30% lower relative risk of a doubling of the creatinine level or death from renal causes in the canagliflozin compared to the placebo group (85). Later findings unequivocally showed that SGLT2i display renoprotective effects regardless of T2D status reducing the relative risk of composite renal outcomes by over 39% (HR 0.61; 95% CI, 0.51 - 0.72; P<0.001) and 28% (HR 0.72; 95% CI 0.64 to 0.82; P<0.001) in the DAPA-CKD and EMPA-KIDNEY trial, respectively (86, 87). Current data thus support a class effect favoring both cardiac and renal events prevention hance playing an imperative role in attenuating the residual risk of T2D (88).

## Concluding remarks

Residual cardiovascular risk in individuals with T2D remains an unresolved matter that needs to be adequately addressed. Hypertension affects a significant majority (eight in ten) of individuals with T2D, amplifying the risk of adverse outcomes. However, the utilization of SGLT2i therapy has shown remarkable potential in attenuating this risk and current evidence supports that a part of this benefit is mediated by the influence of SGLT2i on hypertension through mechanisms that were revised in this article. To effectively combat the adverse consequences of residual cardiovascular risk in individuals with T2D, it is imperative to address the knowledge gaps hereby highlighted and implement targeted interventions.

## Author contributions

Idea & Conceptualization: JB, AC-S, TQ. Supervision: AC-S. Original Draft Preparation: JB. Critical review & Editing: JB, AC-S, WN, TQ, AS. All authors contributed to the article and approved the submitted version.
